# 

**Published:** 1894-02-03

**Authors:** 


					the hospital. - \ February 3rd, 1894]
WITH EXTRA NURSING SUPPLEMENT.
Per Annum, 8s. 6d? post free.
Monthly Parts, 6d.; post free, 8d.
Ditto per Annum, 8s? post free.
tRWICH HOSPl TAL
No. 384. Vol. XV. WITH EXTRA NURSING SUPPLEMENT. Saturday,
February 3rd, 1894.

				

